# Pretreatment Level of Red Cell Distribution Width as a Prognostic Indicator for Survival in a Large Cohort Study of Male Laryngeal Squamous Carcinoma

**DOI:** 10.3389/fonc.2019.00271

**Published:** 2019-04-16

**Authors:** Chi-Yao Hsueh, Hui-Ching Lau, Shengjie Li, Lei Tao, Ming Zhang, Hongli Gong, Liang Zhou

**Affiliations:** ^1^Department of Otolaryngology, Eye Ear Nose and Throat Hospital, Fudan University, Shanghai, China; ^2^Shanghai Key Clinical Disciplines of Otorhinolaryngology, Shanghai, China; ^3^Department of Clinical Laboratory, Eye & ENT Hospital, Shanghai Medical College, Fudan University, Shanghai, China

**Keywords:** laryngeal squamous cell cancer, male, red cell distribution width, prognosis, biomarker

## Abstract

**Objective:** High levels of red cell distribution width (RDW) may be associated with adverse outcomes in patients with cancer. The purpose of the present study was to investigate the prognostic impact of pretreatment RDW levels on overall survival (OS), cancer-specific survival (CSS), and disease-free survival (DFS) in a large cohort of male laryngeal squamous cell cancer (LSCC) patients.

**Methods:** A total of 809 LSCC patients who were treated between 2007 and 2011 at the Eye & ENT Hospital of Fudan University were enrolled and evaluated retrospectively. OS, CSS, and DFS were analyzed using the Kaplan–Meier method. To evaluate the prognostic significance of RDW levels, univariate, and multivariate Cox analyses were applied.

**Results:** Higher pretreatment RDW levels were significantly associated with high death events, red blood cell count, hemoglobin, radiotherapy, operation therapy, and advanced tumor stage (*p* < 0.05). From the univariate analysis, we observed that the higher (13.2–13.5%) and the highest (>13.5%) quartiles of RDW level were consistent factors for poor OS, CSS, and DFS in LSCC patients. In the multivariate analysis, after adjusting for confounding factors, the higher and highest quartiles of RDW levels were identified as independent prognostic factors in male LSCC patients.

**Conclusion:** Higher pretreatment RDW levels were demonstrated to be associated with poor clinical outcome in male LSCC patients and might be novel markers for patient stratification in LSCC management.

## Introduction

Laryngeal cancer is one of the most common cancers of the head and neck, of which the estimated crude incidence and mortality rates in China were 1.86/100,000 and 1.01/100,000, respectively (1). This disease has a male predominance with a male-to-female ratio of 20 to 30:1 in China (2). The most commonly observed histological type of laryngeal cancer is laryngeal squamous cell cancer (LSCC), accounting for 95% of cases involving the stratified squamous epithelial lining of the larynx (3, 4). Despite improvements in diagnosis and treatment in the last four decades, there was an absence of a significant change in the 5-year overall survival (OS) rate for larynx cancer patients (5). Several prognostic factors have been identified to predict prognosis in LSCC patients, such as tumor size, histological subtype or grade, vascular invasion, and lymph node metastases. However, the majority of these factors can only be assessed after surgery.

Although “omics”-based technology has enhanced our perception of possible risk factors, prognoses and/or responses to treatment biomarkers, the validation of novel molecular biomarkers is associated with high costs, time-consuming procedures, and laboratory efforts. Therefore, a simple, rapid, reliable, and cheap pretreatment prognostic marker for LSCC patients is desired. In recent years, many studies have presented red cell distribution width (RDW) as a strong and independent risk factor for death (6, 7). RDW reflects impaired erythropoiesis and abnormal red blood cell survival, while the heterogeneity of red blood cell size correlates with inflammation and undernutrition status (8–10). Recent studies have also shown that RDW could be a prognostic factor in several carcinomas, including ovarian carcinoma (11), lung carcinoma (12), gastric carcinoma (13), and laryngeal carcinoma (14). In a retrospective, observational cohort study including 654 patients with epithelial ovarian cancer, enhanced RDW was found to be significantly associated with poorer overall survival (OS) (11). Bozkurt et al. (14) reported that laryngeal cancer patients (*n* = 132) with high RDW at diagnosis were at a higher risk of locoregional recurrence (hazard ratio [HR] = 5.818, 95% confidence interval (95% CI) 1.25–26.97; *p* = 0.024). Therefore, this study aims to evaluate the prognostic significance of pretreatment RDW levels on disease-free survival (DFS), OS and cancer-specific survival (CSS) in a large cohort of 809 LSCC patients.

## Materials and Methods

### Study Population

This study was conducted in accordance with the Helsinki Declaration and was approved by the committee of the Eye & ENT Hospital of Fudan University, Shanghai, China. Written informed consent was obtained and approved for all patients. The patient information was anonymized and deidentified prior to analysis. The patients were recruited from the Department of Otolaryngology-Head and Neck Surgery at the Eye & ENT Hospital of Fudan University using a primary cohort of consecutive patients who underwent partial or total laryngectomy between January 1, 2007 and December 31, 2011 as their first curative treatment option. All patients were followed up through telephone messages, and outpatient records were obtained every 3 months during the first 2 years and every 6 months thereafter until death events occurred. Survival status, disease progression, and period of death or metastasis were recorded. The last follow-up date was September 30, 2016. DFS was recorded from the date of laryngectomy to the date of recurrence within the follow-up period. CSS was recorded from the date of surgery until death because of intercurrent disease within the follow-up period. OS was recorded from the date of surgery until death.

### Inclusion Criteria

**Figure 1:**
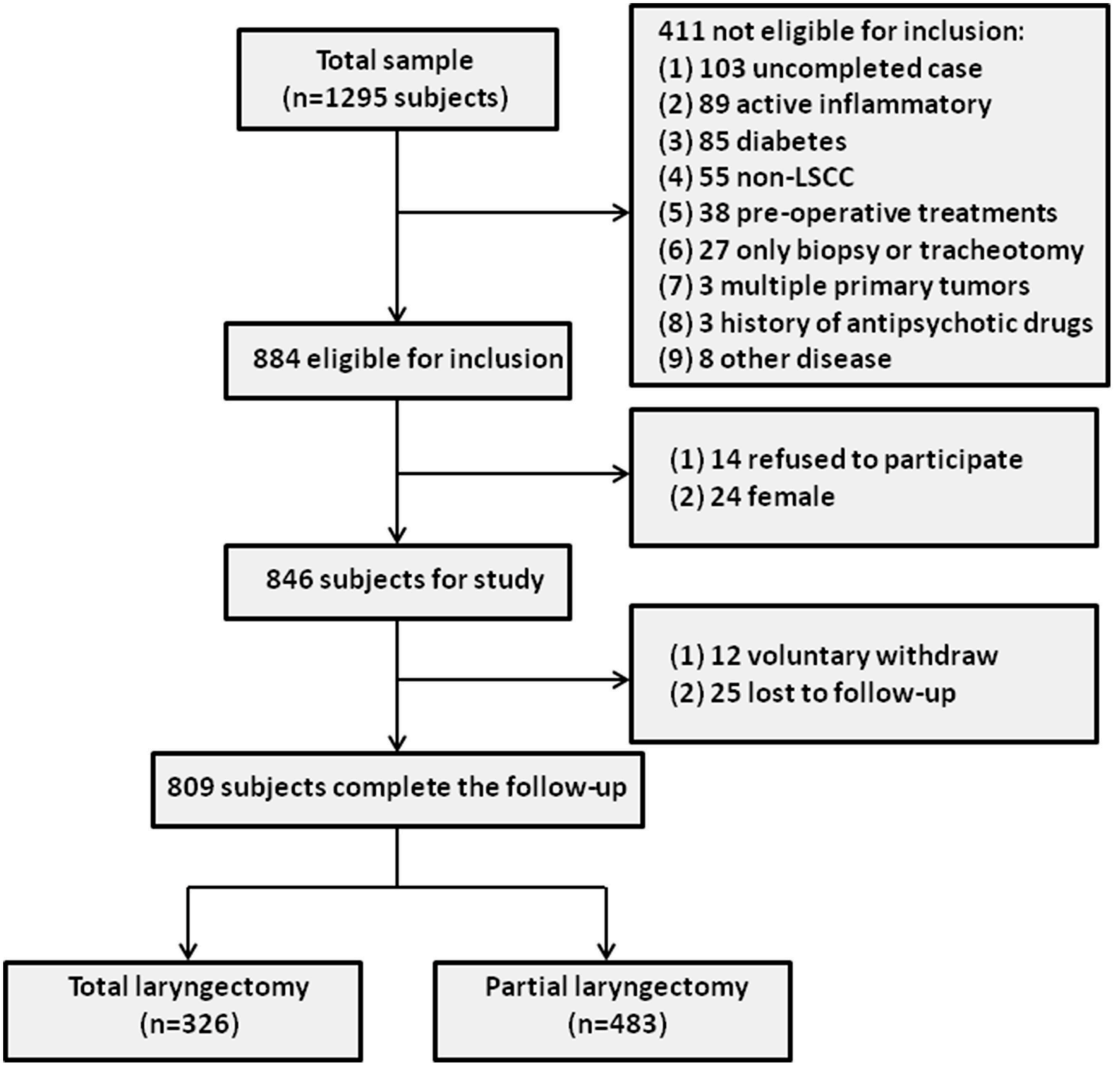
The study cohort flow diagram.

To select the LSCC group, 1295 LSCC patients who visited the Department of Otolaryngology-Head and Neck Surgery at the Eye & ENT Hospital of Fudan University between January 1, 2007 and December 31, 2011 were enrolled. A total of 1,244 subjects completed follow-up, of whom 360 patients were later excluded from the study based on the inclusion criteria. Furthermore, 24 female LSCC subjects were excluded, and 14 LSCC subjects refused to participate in this study. During the follow-up period, 37 LSCC patients (voluntary withdrawal = 12, no response = 25) were excluded, leaving a final sample of 809 LSCC patients. The study cohort flow diagram is shown in Figure 1.

The inclusion criteria and selection process for the included LSCC patients were as follows (15): (1) All patients had histologically proven squamous cell carcinoma, confirmed by pathology and classified under the seventh edition of the TNM-UICC/AJCC stage classification; (2) The patients were a minimum of 18 years old; (3) Blood samples were collected before the patient pretreatment; (4) complete clinical, laboratory, imaging, and follow-up data were collected; (5) LSCC subjects were selected from inpatients; and (6) the subjects were free of systemic diseases (self-reported), such as acute infectious diseases, active inflammation (WBC>10^*^10^9^/l or CRP>10 mg/l),autoimmune disease, and other cancers.

### Data Collection

Clinical and demographic information were obtained from the medical data platform of the Eye & ENT Hospital by two independent investigators. Thereafter, all data were collated by the two investigators together. During this process, the two independent investigators consulted the medical data platform to resolve any possible bias. These data included the following demographic information: age, sex, body mass index, drinking habits, smoking habits, blood pressure, white blood cell levels, red blood cell count, red blood cell distribution width levels, hemoglobin levels, creatinine levels, nitrogen levels, aspartate transaminase levels, and alanine aminotransferase levels. The following clinical information was also collected: medical history, date of diagnosis, tumor subsite, tumor size, local, and regional extension category of the primary tumor, clinical stage, differentiation grade, prior surgical therapy, and level of neck dissection. The follow-up data included date of primary resection, date and type of relapse, date of diagnosis of metastatic disease, and date of death.

Blood samples for routine blood examination were collected via standard venipuncture of the veins in the antecubital fossae (anterior elbow veins). White blood cell levels, red blood cell count, red blood cell distribution width levels, and hemoglobin levels were measured with the Mindray BC-5500 (Shenzhen, China) automatic blood counting system within 0.5 h after blood collection. Blood samples for biochemical measurements were also collected. Serum levels of creatinine, nitrogen, aspartate transaminase, and alanine aminotransferase were measured using a commercially available kit (Roche Diagnostics GmbH, Mannheim, Germany).

### Statistical Analysis

All analyses were performed using SPSS 13.0 software (SPSS Inc., Chicago, IL). Normality was assessed using the Kolmogorov–Smirnoff test. RDW was divided into quartiles in order to fully cover the non-linearity. The association between the RDW levels and clinicopathological parameters was evaluated by one-way ANOVA and a chi-square test. Univariate Cox regression analyses were performed to determine the association between RDW on OS, DFS, and CSS. After application of the univariate Cox regression analysis, a multivariate Cox regression analysis (adjusted for covariates) was used to analyze the association between RDW and the OS, DFS, and CSS of LSCC. The patient clinical end points were calculated using the Kaplan–Meier method and compared by the log-rank test. Hazard ratios (HR) estimated from the Cox analysis were reported as relative risks with corresponding 95% confidence intervals (CI). A two-sided *p* < 0.05 was considered statistically significant.

## Results

A total of 809 male LSCC subjects were eligible in this study. During a mean longitudinal follow-up period of 72.9 months (from a range of 3 to 116 months) after the surgery, 186 deaths events occurred (23.0%). The mean age of the participants was 60.6 years. Only 30.28% of the participants reported never smoking, and 62.1% reported never drinking. The baseline characteristics and clinicopathological features of the study subjects are shown in Table 1.

**Table 1:** Baseline demographic and lifestyle characteristics of laryngeal squamous cell cancer patients.

**Covariates**	**Number of Patients/mean**
No. individuals	809
Age at diagnosis (years), mean, range	60.63, 27–89
≤40	15
40–60	416
≥60	378
BMI (kg/m^2^), mean, range	23.42, 16.72–28.43
Male (%)	100%
Death events	186
Duration of follow-up, mean, range (months)	72.89, 3–116
Smoking (yes/no)	565/244
Drinking (yes/no)	307/462
Hypertension (yes/no)	226/583
Tumor recurrence, mean, range (months)	65, 1–116
**POSTOPERATIVE RADIOTHERAPY AND CHEMOTHERAPY**	
Radiotherapy	140
Chemotherapy	28
None	641
**T STAGE**	
T_1_	195
T_2_	325
T_3_	237
T_4_	52
**N STAGE**	
N_0_	718
N_1_	31
N_2−3_	60
**TNM STAGE**	
I	195
II	300
III	214
IV	100
**HISTOLOGICAL DIFFERENTIATION**	
Highly or moderately differentiated	670
Poorly differentiated	22
Not available	117
Tumor size, mean, range (cm)	1.80, 0–6.5
Tumor size (≤2 cm/>2 cm)	511/298
**OPERATION THERAPY**	
Total laryngectomy	326
Partial laryngectomy	483
**TUMOR SUBSITE**	
Supraglottic	177
Glottic	621
Subglottic	11
WBC, mean, range (10^9^/l)	6.24, 3.05–14.6
RBC, mean, range (10^9^/l)	4.29,1.83–6.68
Hg, mean, range(g/l)	133.14, 61–174
RDW, mean, range (%)	13.15, 11.5–18.5
**LIVER FUNCTION**	
AST, mean, range (u/l)	20.46, 8-114
ALT, mean, range (u/l)	23.28, 4–143
**RENAL FUNCTION**	
BUN, mean, range (mmol/l)	6.00, 2.47–14.18
Cr, mean, range (umol/l)	74.15, 33–209

*RDW, red cell distribution width, Cr, creatinine, BUN, urea nitrogen, AST, aspartate transaminase, ALT, alanine aminotransferase, Hg, hemoglobin, WBC, white blood cell, RBC, red blood cell count, BMI, body mass index*.

To test whether high RDW levels influence the clinical outcome of LSCC patients, we first subdivided the LSCC patients into four groups according to their RDW quartiles. The RDW quartiles were divided into the lowest quartile (RDW ≤ 12.9%), the lower quartile (12.9% < RDW ≤ 13.2%), the higher quartile (13.2% < RDW ≤ 13.5%), and the highest quartile (>13.5%). Rising levels of RDW were significantly correlated with higher death events, radiotherapy, reduced red blood cell count, reduced hemoglobin, long duration of follow-up, drinking habit, operation therapy, and advanced tumor stage (*p* < 0.05), whereas no association with age, smoking habits, hypertension, chemotherapy, tumor histological grade could be found (*p* > 0.05) (Table 2).

**Table 2:** The relation between clinicopathological parameters and pre-treatment red cell distribution width levels of patients with laryngeal squamous cell cancer.

	**RDW quartiles**					
	**≤12.9% (*n* = 242)**	**12.9 −13.2% (*n* = 244)**	**13.2–13.5% (*n* = 179)**	**>13.5% (*n* = 144)**	***Chi-Square/t value***	***P*-Value for Trend**
Age (years)	60.42	60.89	60.60	60.58	0.094	0.963
	40–89	31–84	30–89	27–85		
Death events	25	37	60	64	45.333	< 0.001
Duration of follow-up	78.84	73.87	69.81	64.84	7.393	< 0.001
	6–115	3–115	12–114	7–116		
Smoking	178	174	121	92	0.826	0.363
Drinking	98	81	69	59	0.026	0.871
Hypertension	64	75	50	37	0.025	0.875
RBC	4.35	4.33	4.22	4.22	2.173	0.038
Hg	137.50	132.15	131.31	131.27	10.173	< 0.001
Radiotherapy	32	35	38	35	7.134	0.008
Chemotherapy	5	7	10	6	2.535	0.111
**T STAGE**						
T_1_	37	77	40	41	3.434	0.064
T_2_	163	37	50	75	4.891	0.027
T_3_	24	126	79	8	0.084	0.772
T_4_	18	4	10	20	4.648	0.031
**N STAGE**						
N_0_	242	214	130	132	1.420	0.233
N_1_	0	20	3	8	–	–
N_2−3_	0	10	46	4	–	–
**TNM STAGE**						
I	37	77	40	41	3.434	0.064
II	163	27	39	71	8.069	0.005
III	24	129	53	8	0.769	0.380
IV	18	11	47	24	18.486	< 0.001
**HISTOLOGICAL DIFFERENTIATION**						
Highly or moderately differentiated	207	199	149	115	0.145	0.703
Poorly differentiated	5	5	11	1	0.137	0.712
**OPERATION THERAPY**						
Total laryngectomy	66	109	97	54	4.599	0.032
Partial laryngectomy	176	135	82	90	8.569	0.036

*RDW, red cell distribution width; Hg, hemoglobin; WBC, white blood cell; RBC, red blood cell count; BMI, body mass index*.

Pearson correlation analyses were performed to identify the associations between RDW and hemoglobin. A significant negative correlation was found between the RDW and hemoglobin (*r* = −0.186, *p* < 0.001).

To investigate whether RDW level could be associated with the clinical outcome of LSCC patients, univariate, and multivariate Cox proportional models for OS, CSS, and DFS were calculated. Univariate analysis identified high RDW as a poor prognostic factor for OS (*p* < 0.001), CSS (*p* < 0.001), and DFS (*p* < 0.001) in this study cohort (Table 3).

**Table 3:** Univariate Cox regression analysis for overall survival, disease-free survival and cancer specific survival in patients with laryngeal squamous cell cancer.

	**OS**		**DFS**		**CSS**	
	**HR (95%CI)**	***P***	**HR (95%CI)**	***P***	**HR (95%CI)**	***P***
**RDW**						
≤12.9						
12.9–13.2	1.41 (0.866–2.305)	0.167	1.63 (0.982–2.697)	0.059	1.72 (1.007–2.944)	0.047
13.2–13.5	1.79 (1.433–2.243)	< 0.001	1.91 (1.514–2.409)	< 0.001	1.91 (1.484–2.456)	< 0.001
>13.5	1.67 (1.445–1.939)	< 0.001	1.78 (1.525–2.070)	< 0.001	1.74 (1.477–2.057)	< 0.001
**RBC**						
< 4.29	1		1		1	
>4.29	1.009 (0.860–1.183)	0.916	1.028 (0.877–1.206)	0.731	1.010 (0.861–1.184)	0.961
**Hg**						
>133.14	1		1		1	
< 133.14	1.338 (1.141–1.570)	< 0.001	1.189 (1.014–1.395)	0.033	1.138 (1.141–1.570)	< 0.001
**RADIOTHERAPY**						
Yes	1		1		1	
No	1.576 (1.124–2.211)	0.008	1.799 (1.283–2.524)	0.001	1.556 (1.109–2.128)	0.010
**CHEMOTHERAPY**						
Yes	1		1		1	
No	1.941 (1.176–3.024)	0.009	2.191 (1.328–3.614)	0.002	1.910 (1.151–3.136)	0.012
**TNM STAGE**						
I-II	1		1		1	
III-IV	2.714 (2.033–3.621)	< 0.001	2.882 (2.161–3.845)	< 0.001	3.107 (2.291–4.212)	< 0.001
**T STAGE**						
T_1−2_	1		1		1	
T_3−4_	2.392 (1.798–3.176)	< 0.001	2.532 (1.906–3.366)	< 0.001	2.726 (2.026–3.673)	< 0.001
**N STAGE**						
N_0_						
N_1_	1		1		1	
N_2−3_	3.254 (2.124–4.785)	< 0.001	3.408 (2.317–5.010)	< 0.001	3.435 (2.320–5.13)	< 0.001
**TUMOR SUBSITE**						
Glottic and Subglottic	1		1		1	
Supraglottic	2.250 (1.654–3.003)	< 0.001	2.380 (1.774–3.195)	< 0.001	2.127 (1.654–2.967)	< 0.001
**OPERATION THERAPY**						
Partial laryngectomy	1		1		1	
Total laryngectomy	2.472 (1.881–3.255)	< 0.001	2.621 (1.993–3.449)	< 0.001	2.469 (1.877–3.244)	< 0.001
**TUMOR SIZE**						
≤2 cm	1		1		1	
>2 cm	2.650 (1.995–3.524)	< 0.001	2.634 (1.980–3.500)	< 0.001	2.765 (2.080–3.677)	< 0.001
**AGE**						
< 60	1		1		1	
≥60	1.676 (1.250–2.245)	0.001	1.595 (1.190–2.136)	0.002	1.569 (1.160–2.122)	0.003
**BMI**						
< 23.42	1		1		1	
≥23.42	0.861 (0.738–1.134)	0.356	0.865 (0.645–1.234)	0.322	0.889 (0.679–1.167)	0.336
**HYPERTENSION**						
No	1		1		1	
Yes	1.254 (0.925–1.697)	0.144	1.302 (0.963–1.766)	0.093	1.219 (0.888–1.673)	0.225
**SMOKING HISTORY**						
No	1		1		1	
Yes	1.057 (0.774–1.447)	0.728	1.103 (0.805–1.508)	0.539	1.105 (0.796–1.537)	0.548
**DRINKING HISTORY**						
No	1		1		1	
Yes	1.111 (0.833–1.484)	0.474	1.162 (0.871–1.554)	0.309	1.139 (0.844–1.539)	0.396

*RDW, red cell distribution width; Hg, hemoglobin; WBC, white blood cell; RBC, red blood cell count; BMI, body mass index*.

To determine the independent prognostic value of the RDW levels for OS, CSS, and DFS, a multivariate analysis using a Cox proportional hazard model was performed. After adjustments for age, body mass index, smoking, drinking, hypertension, white blood cell, hemoglobin, red blood cell count, creatinine, nitrogen, aspartate transaminase, alanine aminotransferase, tumor size, histological grade, tumor stage, tumor subsite and operation therapy, we identified the RDW level within the higher quartile (HR = 1.445, 95% CI = 1.721–2.894, *p* < 0.001) and the highest quartile (HR = 2.047, 95% CI = 1.587–2.641, *p* < 0.001) as independent prognostic factors for OS (Table 4); the RDW level within the higher quartile (HR = 1.339, 95% CI = 1.105–2.774, *p* < 0.001) and the highest quartile (HR = 2.043, 95% CI = 1.584–2.635, *p* < 0.001) as independent prognostic factors for CSS (Table 4); and the RDW level within the higher quartile (HR = 1.539, 95% CI = 1.787–2.010, *p* < 0.001) and the highest quartile (HR = 2.089, 95% CI = 1.622–2.691, *P* < 0.001) as independent prognostic factors for DFS (Table 4).

**Table 4:** Multivariate Cox regression analysis for overall survival, disease-free survival and cancer specific survival in patients with laryngeal squamous cell cancer.

	**OS**		**DFS**		**CSS**	
	**HR (95%CI)**	***P***	**HR (95%CI)**	***P***	**HR(95%CI)**	***P***
**RDW**						
≤12.9	1		1		1	
12.9–13.2	1.216 (0.374–3.957)	0.745	1.223 (0.375–3.989)	0.739	1.339 (0.410–4.367)	0.629
13.2–13.5	1.445 (1.721–2.894)	< 0.001	1.539 (1.787–2.010)	< 0.001	1.339 (1.105–2.774)	< 0.001
>13.5	2.047 (1.587–2.641)	< 0.001	2.089 (1.622–2.691)	< 0.001	2.043 (1.584–2.635)	< 0.001
**Hg**						
>133.14	1		1		1	
≤133.14	1.009 (0.721–1.412)	0.945	0.959 (0.685–1.343)	0.809	1.005 (0.718–1.407)	0.975
**RADIOTHERAPY**						
Yes	1		1		1	
N0	1.151 (0.752–1.763)	0.518	1.331 (0.869–2.038)	0.189	1.143 (0.746–1.750)	0.539
**CHEMOTHERAPY**						
Yes	1		1		1	
N0	1.497 (0.751–2.985)	0.252	1.323 (0.661–2.647)	0.429	1.50 (0.752–2.991)	0.249
**TNM STAGE**						
I-II	1		1		1	
III-IV	1.517 (0.657–3.503)	0.329	1.416 (0.607–3.307)	0.421	1.510 (0.651–3.465)	0.341
**T STAGE**						
T_1−2_	1		1		1	
T_3−4_	1.097 (0.514–2.338)	0.811	1.147 (0.534–2.467)	0.725	1.101 (0.517–2.346)	0.802
**N STAGE**						
N_0_						
N_1_	1		1		1	
N_2−3_	1.271 (0.696–2.324)	0.435	1.143 (0.618–2.112)	0.670	1.279 (0.700–2.339)	0.423
**TUMOR SUBSITE**						
Glottic and Subglottic	1		1		1	
Supraglottic	0.808 (0.560–1.166)	0.255	0.884 (0.581–1.227)	0.375	0.808 (0.560–1.166)	0.255
**OPERATION THERAPY**						
Partial laryngectomy	1		1		1	
Total laryngectomy	1.404 (0.930–2.120)	0.106	1.538 (1.017–2.325)	0.041	1.042 (0.928–2.116)	0.108
**TUMOR SIZE**						
≤2 cm	1		1		1	
>2 cm	1.251 (0.806–1.944)	0.318	1.251 (0.802–1.953)	0.324	1.256 (0.809–1.952)	0.310
**AGE**						
< 60	1		1		1	
≥60	1.732 (1.248–2.405)	0.001	1.579 (1.133–2.199)	0.007	1.735 (1.249–2.409)	0.001

*RDW, red cell distribution width; Hg, hemoglobin. Adjusted for age, body mass index, smoking, drinking, hypertension, white blood cell, hemoglobin, red blood cell count, creatinine, nitrogen, aspartate transaminase, alanine aminotransferase, tumor size, histological grade, tumor stage, tumor subsite, and operation therapy*.

**Figure 2:**
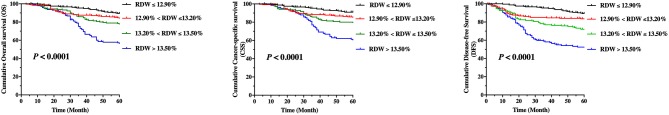
Kaplan–Meier curve stratified by red cell distribution width according to quartiles regarding overall survival (OS), cancer-specific survival, and disease-free survival (DFS) for patients with laryngeal squamous cell cancer.

Kaplan–Meier curves for OS, CSS, and DFS, which comprise groups according to quartiles of the RDW levels, are shown in Figure 2. The pairwise log-rank test indicates significant differences between the highest quartile (RDW > 13.5%) compared with the lowest quartile (RDW ≤ 12.9%), the lower quartile (13.2% ≥ RDW > 12.9%), and the higher quartile (13.5% ≥ RDW > 13.2%). The 5-year OS rates for patients in the RDW quartiles were as follows: the lowest quartile (89.3%), the lower quartile (84.6%), the higher quartile (78.3%), and the highest quartile (57.1%) (*p* < 0.001, Figure 2). The 5-year CSS rates for patients in the RDW quartiles were: the lowest quartile (91.0%), the lower quartile (85.75%), the higher quartile (80.08%), and the highest quartile (61.3%) (*P* < 0.001, Figure 2). The 5-year DFS rate for patients in the RDW quartiles were: the lowest quartile (89.3%), the lower quartile (83.5%), the higher quartile (72.0%), and the highest quartile (52.5%) (*p* < 0.001, Figure 2). Kaplan–Meier curves for OS, CSS, and DFS reveals that a high RDW level could be a risk factor consistent with poor prognosis in LSCC patients (*p* < 0.001, log-rank test).

## Discussion

In this large-scale, retrospective study, we demonstrated that high RDW levels at the time of pretreatment were associated with adverse OS, CSS, and DFS in LSCC patients. Our multivariate analyses have shown that elevated pretreatment RDW is a risk factor, independent from other parameters, including age, smoking, drinking, TNM stage, tumor location, and surgery methods, and could be a useful hematological marker for poor prognosis prediction.

Inflammation and oxidative damage foster multiple cancer hallmark functions, including sustaining proliferative signaling, evading growth suppressors, resisting cell death, enabling replicative immortality, inducing angiogenesis, and activating invasion and metastasis, thus impacting patient survival (16). Elevated RDW has been reported to be a biomarker of inflammation and oxidative stress (17, 18). However, the prognostic significance of RDW in LSCC remains unknown.

Many efforts have been made to investigate the relationship between RDW and prognosis in various types of cancer. In other cancer studies, rich RDW levels have already been reported to be associated with poor prognosis and recurrence. For example, a meta-analysis of six studies predominantly conducted in a retrospective design showed that elevated RDW was significantly associated with worse OS/CSS of esophageal cancer patients when RDW > 13% (19). Moreover, a meta-analysis of eight cohort studies showed that elevated RDW may be an indicator of poor prognosis in upper aerodigestive tract cancers (20). Albayrak et al. (21) performed a case-control study and showed that higher RDW was associated with an increased risk of prostate cancer progression. Życzkowski et al. (22) retrospectively evaluated 434 patients and showed that RDW might be an easily obtainable prognostic marker in renal cell carcinoma patients treated with nephrectomy.

Only five studies have examined the relationship of RDW with cancer in the head and neck region, of which one study was for the thyroid cancer (23), two were for cancers of the oral cavity (24, 25) and two were for laryngeal cancers (14, 26). For thyroid cancer, Aktas et al. (23) reported that the level of RDW was significantly higher compared with that in normal subjects. Regarding oral cancer, the previously reported data were conflicting. Ge et al. (25) reported, in 236 patients, that an elevated preoperative RDW (≥15%) during diagnosis may independently predict poorer OS in patients with oral squamous cell carcinoma, while Tangthongkum et al. (24) found that RDW has no prognostic value on any outcome in patients with oral cancer. For laryngeal cancer, Kara et al. (26) reported in a smaller (*n* = 81) study and Bozkurt et al. (14) reported in 132 patients that RDW was an independent prognostic factor of disease survival. Moreover, in a smaller study, including 205 patients with head and neck cancer, Tham et al. (27) reported that a low hemoglobin/RDW ratio was associated with poorer event-free survival (HR = 2.02, *p* = 0.017). However, many of these studies included rather small numbers of investigated cases and differed in terms of inclusion criteria and clinical end points. In our study, we validated the prognostic impact of RDW levels on OS, CSS, and DFS as the end points and clearly demonstrated that the pretreatment RDW level was independently associated with OS, CSS, and DFS in a large cohort of 809 LSCC patients.

The possible role of RDW in the development of cancers has not been previously elucidated in experimental settings. Several factors could explain the prognostic value of RDW. The accepted explanation is that a higher tumor stage can result in a greater extent of systemic inflammation by the secretion of cytokines and the release of tumor-degradation products (28), which then decreases the lifetime of red blood cells (29) and increases the RDW level. Moreover, patients with advanced stage cancers might change their dietary habits or eat less due to problems with swallowing, which might lead to malnutrition and thereafter, a decreased volume of red blood cells, especially RDW levels, before their operations (30).

We must admit that several limitations in our study are as follows: RDW is associated with systematic inflammation but is not a specific marker of inflammation. Although we excluded interference factors, such as acute infectious diseases, and system diseases, chronic subclinical inflammation in LSCC subjects is still difficult to detect through medical screenings. Thus, there is a possibility to influence the RDW level. Furthermore, nutritional issue may be a confounding factor, but we were unable to evaluate this feature in our study. Additionally, our study is limited by its retrospective nature: the data were collected at a single-center in a retrospective study with a homogeneous group of single genders, which might limit the generalizability of the results.

In conclusion, excluding human bias, a high level of pretreatment RDW may be a poor prognostic factor for survival in male LSCC patients. Further studies should attempt to illuminate the mechanism and confirm its role in different population groups.

## Ethics Statement

This study was conducted in accordance with the Helsinki Declaration and was approved by the committee of the Eye & ENT Hospital of Fudan University, Shanghai, China. Written informed consent was obtained and approved for all patients.

## Author Contributions

HG, C-YH, and LZ designed the study. HG, C-YH, MZ, LT, and LZ contributed to the patient recruitment and collected the data. H-CL and C-YH performed the statistical analysis. H-CL and C-YH wrote the manuscript. All authors read and approved the final manuscript.

### Conflict of Interest Statement

The authors declare that the research was conducted in the absence of any commercial or financial relationships that could be construed as a potential conflict of interest.
